# Gene Dosage Analysis on the Single-Cell Transcriptomes Linking Cotranslational Protein Targeting to Metastatic Triple-Negative Breast Cancer

**DOI:** 10.3390/ph14090918

**Published:** 2021-09-10

**Authors:** Yining Liu, Min Zhao

**Affiliations:** 1 The School of Public Health, Institute for Chemical Carcinogenesis, Guangzhou Medical University, Guangzhou 511436, China; yiningliu.pku@gmail.com; 2 School of Science, Technology and Engineering, University of the Sunshine Coast, Maroochydore, QLD 4558, Australia

**Keywords:** single-cell RNA sequencing, copy number variation, signal recognition particle, dosage effect, metastatic triple-negative breast cancer

## Abstract

Many recent efforts have been put into the association between expression heterogeneity and different cell types and states using single-cell RNA transcriptome analysis. There is only limited understanding of gene dosage effects for the genetic heterogeneity at the single-cell level. By focusing on concordant copy number variation (CNV) and expression, we presented a computational framework to explore dosage effect for aggressive metastatic triple-negative breast cancer (TNBC) at the single-cell level. In practice, we collected CNV and single-cell expression data from the same patients with independent technologies. By focusing on 47,198 consistent copy number gains (CNG) and gene up-regulation from 1145 single cells, ribosome proteins with important roles in protein targeting were enriched. Independent validation in another metastatic TNBC dataset further prioritized signal recognition particle-dependent protein targeting as the top functional module. More interesting, the increased ribosome gene copies in TNBC may associate with their enhanced stemness and metastatic potential. Indeed, the prioritization of a well-upregulated functional module confirmed by high copy numbers at the single-cell level and contributing to patient survival may indicate the possibility of targeted therapy based on ribosome proteins for TNBC.

## 1. Introduction

Triple-negative breast cancer (TNBC) is characterized by none of the cell surface receptors for estrogen, progesterone, or human epidermal growth factor receptor 2 (HER2) [1]. Because of this, TNBC cannot be treated by simply blocking these receptors. Although current treatment for TNBC has mainly relied on the response to chemotherapy, the potential excessive side effects may cause the treatment to fail. Therefore, identification and understanding of TNBC have a profound influence on the development of effective drugs for cancer therapies. Several large-scale cancer genomics projects focused on identifying novel receptors for complementary treatment [2].

Tumor tissue usually consists of multiple cell types, including different carcinoma subclones other than pure carcinoma cells. Recent advances in single-cell sequencing technology enable the characterization of spatiotemporal features for thousands of cells simultaneously [3]. The single-cell-based transcriptome analyses mostly rely on cell type-specific marker genes. Although the precise identification of the cell types is important, many common genetic features and molecular consequences across different cell types may be overlooked during cancer development.

In the human genome, a copy number variation (CNV) is defined by the number of copies of a particular DNA fragment, which varies in a population. These gains and losses of genetic material may contribute to human disease by gene dosage effect, which means changing the gene expression by CNV. It has long been recognized that an elevated copy number could favor the progression of an oncogene towards cancer development [4] while a loss of copy number may deactivate tumor suppressors [5].

Although sensitivity of particular gene expressions to CNV has been detected through tissue-based bulk sequencing, the cell type identification-based single-cell transcriptome approach may overlook some significant CNV signatures across different cell types [6]. In addition, the common and profound dosage effects on gene expression across different cell types are largely unknown. Since marker gene-based expression signatures are mainly used to characterize cell states such as metastasis and stemness, the CNV-expression analysis may allow for zooming in on genetic diversity and molecular consequences. To fill these knowledge gaps, we conducted a cross-cell type CNV analysis with matched single-cell transcriptomes to cross-validate and better elucidate the commonality of gene dosage effect in multiple cell types for TNBC.

## 2. Results

To survey the dosage effects of CNVs at the single-cell level, we constructed a computational framework to examine gene expression and copy number variation. By applying the computational framework to two independent metastatic TNBC single-cell transcriptome datasets, we identified the common functional modules for further functional and clinical evaluation.

### 2.1. The Computational Framework to Characterize the Concordant Copy Number Variation and Gene Expression Changes at the Single-Cell Level

To save money, many CNVs were inferred based on single-cell RNA sequencing (scRNASeq) expression data, which are not independent and not suitable for cross-validation. Theoretically, we required the input CNV data, and single-cell transcriptomes should have been derived from a different technological platform and paired to each other at the patient level. For example, the bulk sequencing for all the DNA content from a tumor sample of one patient and the scRNASeq could be independently applied to thousands of cells from the same sample.

**Figure 1:**
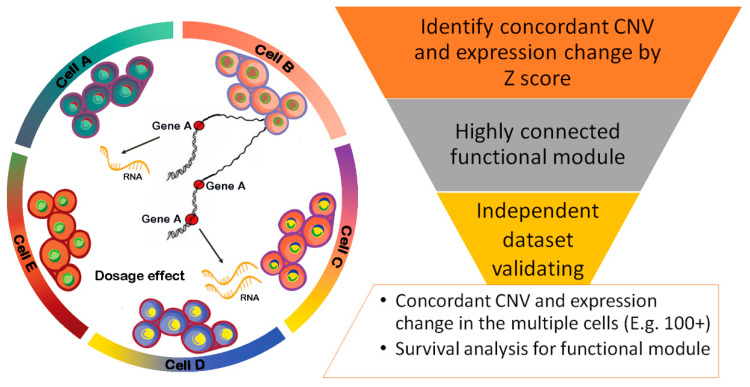
The workflow to explore the global CNV patterns across multiple cell types with gene expression changes at the single-cell level. This workflow starts from the CNV and expression profiling at the single-cell level from the same tissue samples. By mapping the CNV and gene expression changes in the same cells, this workflow will identify concordant CNV and expression changes. By running the gene–gene interaction network analysis, the workflow will build highly connected functional modules to prioritize the key genes with significant gene dosage effects. We also recommend validating the whole process by integrating other independent datasets with both CNV and expression data at the single-cell level. The final validated functional modules in multiple cells will be highly reliable for further clinical feature evaluation and experimental validation.

As shown in Figure 1, our pipeline started with a compilation of matched CNV data from DNA sequencing and expression data from single-cell RNA sequencing. Next, we transformed the cell-based RNA expression profile to the Z-score, which depicts how genes were expressed relatively higher or lower in a particular cell compared to those of the quantitative expression values from other cells. Due to the limitation of RNA content, scRNAseq often has a lot of dropouts, which results in none of the RNA short reads being detected in scRNAseq. To prevent nonsense Z-scores, we masked those cells without expression values. To extract a meaningful Z-score for further mapping to CNV data, we set a threshold for the absolute Z-score of over 1.96, which means significant *p*-values 0.05. Then, the transformed significant Z-score for each gene in a particular cell was mapped to the CNV data from the same patient. The concordant copy number events were screened based on the criteria of whether the copy number gain/loss in the cell correlated with a consistent gene up/downregulation. By counting how many consistent CNV-expression events occurred across all the cells from the input dataset, we further inferred a list of genes with recurrent CNV-based dosage changes. By incorporating the known gene–gene interaction and functional similarity, the framework identified functional modules for those recurrent CNV-changed genes detected in hundreds of cells regardless of their cell types.

In summary, the input for our framework was the independent CNV and scRNAseq data from the same patients. The output was a connected functional module that comprised the top mutated genes based on consistent CNV and expression changes. To further reduce the false positives of functional modules inferred from our computational pipeline, we suggest validating those functional modules in another independent dataset. Once the top prioritized functional modules and genes are confirmed, any routine computational or experimental design can be adopted to further explore the significance. For instance, our final step of this study was to take advantage of large-scale cancer genomics data to evaluate whether those top-ranked genes were highly mutated and associated with patient survival.

### 2.2. The CNG-Driven Increased Gene Expression in Multiple TNBC Cell Types

To focus on TNBC and explore the potential novel targets for cancer therapy, we applied this computational pipeline to the TNBC dataset GEO118390 with six patients and an expression profile from 1533 cells. Instead of cell-type identification, we focused on the common mechanisms underlying copy number variants across all the cell types. To avoid expression bias on those longer genes with more abundant sequencing reads, we used transcripts per million (TPM) as the quantification method. Specifically, the sum of all TPMs in all the 1533 cells is the same, which makes the proportion of short reads mapped to a gene comparable in different cells.

By starting from a TPM expression matrix with 21,785 genes × 1533 cells, we first generated the same size matrices with 33,396,405 Z-scores (21,785 rows and 1533 columns), which depicted the relative expression of a gene over the averaged expression rate across all the 1533 cells. A positive Z-score means the gene expression is relatively higher than the expressions from the remaining cells. By running through the described computational pipeline, we first masked 29,931,625 zero expression values. Based on the remaining 3,464,780 meaningful Z-scores, we mapped the CNV data from the whole exome sequencing in the same six patients. In total, 1,440,802 non-redundant CNV-expression events in 1245 cells were obtained.

However, most CNV-expression events (1,351,278) may not be informative since these expression variations are not statistically meaningful (e.g., their absolute Z-score < 1.96). In contrast, we defined 89,524 meaningful CNV-expression events as consistent events with an absolute Z-score cutoff over 1.96 (Table S1). On the other hand, some CNV-expression consistency only occurred in a small number of cells. By pulling out all the cells with gains or losses of gene copies, we set a threshold of 100 or more cells to prioritize the most informative copy number variation events. Notably, we only focused on the consistent copy number gain and gene upregulation (CNG-UP) genes because the majority of the Z-scores for downregulation were higher than −1.96. Finally, these step-by-step filterings identified a total of 47,514 CNG-UP events associated with 94 genes across 1145 cells. It is worth noting that these consistent patterns across patients are highly reliable, which may empower scRNASeq-driven clinical decisions in future.

**Figure 2:**
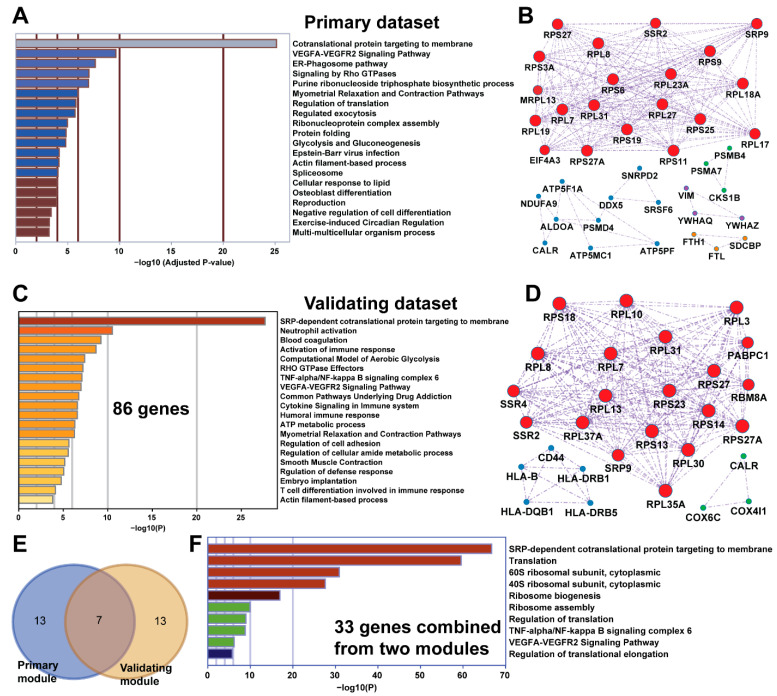
Functional analysis of two top-ranked gene lists with concordant copy number gain and upregulation (CNG-UP) genes. (**A**) The bar plot shows the gene ontology (GO) cluster representatives for the 94 CNG-UP genes from the primary scRNAseq dataset. The *x*-axis indicates the log of corrected *p*-values. (**B**) The five functional modules-based gene–gene interactions for the 94 CNG-UP genes from the primary scRNAseq dataset. The different colors represent the five identified functional modules. The top ranked modules related to cotranslational protein targeting membranes are depicted in red nodes. Four other identified modules are purine ribonucleoside triphosphate biosynthetic processes (blue nodes), SCF(Skp2)-mediated degradation of p27/p21 (green nodes), apoptosis (purple nodes), and neutrophil degranulation (orange nodes). (**C**) The representative GO clusters for 86 CNG-UP genes from the validating dataset. The *x*-axis indicates the log of corrected *p*-values. (**D**) Three functional modules summarized from gene–gene interactions for the 86 CNG-UP genes from the validating dataset. The top three ranked modules are SRP-dependent cotranslational protein targeting to membranes (in red), the interferon-gamma-mediated signaling pathway (in blue), and cellular responses to stress (in green). (**E**) Overlapping of the top-ranked modules associated with protein targeting from primary and validating datasets. Seven genes are shared in both modules. (**F**) The representative GO clusters for 33 CNG-UP genes related to SRP-dependent cotranslational protein targeting to membranes combined from the primary and validating datasets. The *x*-axis indicates the log of corrected *p*-values.

To obtain a functional overview for those 94 top-ranked genes with amplifications in hundreds of cells, we performed comprehensive functional analysis on gene ontology (GO) and well-annotated biological pathways. As shown in Figure 2A, the most significantly enriched functional term was the cotranslational protein targeting to membranes, associated with 20 genes (corrected *p*-value = 10 × 10^−25.133^), which may also overlap with regulated exocytosis (corrected *p*-value = 10 × 10^−5.709^), protein folding (corrected *p*-value = 10 × 10^−25.133^), and regulation of translation (corrected *p*-value = 10 × 10^−4.847^). Those genes may also have roles in many fundamental cellular processes such as ER-phagosome (corrected *p*-value = 10 × 10^−7.659^), spliceosome (corrected *p*-value = 10 × 10^−4.053^), glycolysis, and gluconeogenesis (corrected *p*-value = 10 × 10^−4.806^). In addition, the genes also take part in multiple signaling pathways associated with VEGFA-VEGFR2 (corrected *p*-value = 10 × 10^−9.642^) and Rho GTPase (corrected *p*-value = 10 × 10^−7.052^). Some of genes may also influence negative regulation of cell differentiation (corrected *p*-value = 10 × 10^−3.409^), purine ribonucleoside triphosphate biosynthetic processes (corrected *p*-value = 10 × 10^−7.002^), and reproduction (corrected *p*-value = 10 × 10^−3.920^). In summary, this quick functional enrichment provides a novel insight into the association between TNBC and protein synthesis, folding, and sorting at the single-cell level.

By mapping those genes to the human interactome, we further identified the functional modules. In practice, the molecular complex detection (MCODE) algorithm [7] was applied to cluster those GO-associated genes into five distinct modules (Table S2). For example, module 1 is a cluster of all the genes from the cotranslational protein targeting to membranes (GO:0006613) and SRP-dependent cotranslational protein targeting to membranes (GO:0006614). Similarly, another four functional modules are represented by purine ribonucleoside triphosphate biosynthetic processes, SCF(Skp2)-mediated degradation of p27/p21, apoptosis, and neutrophil degranulation (Figure 2B). Although these modules confirmed the functional enrichment result, the highly connected complex module also helped us to focus on the most important genes in TNBC development.

To validate our finding in another independent TNBC dataset (GSE75688), we focused on a single-cell transcriptome from five TNBC patients with 130 cells. By integrating 20,651 CNG events and 7,528,950 Z-scores for all the 57,915 genes, we found 705,873 CNV-expression events based on the same cells and patients. By further narrowing down the interesting genes based on Z score (>1.96), we harvested a total of 86 genes with CNG-UP events in 6 or more cells. The consistent CNG-UPs detected in the 6+ cell were mapped to gene–gene interactome data and were used to identify three functional modules. Notably, we confirmed the top ranked functional module, which was SRP-dependent cotranslational protein targeting to membranes (Figure 2C,D). By overlapping those genes identified from both datasets related to protein targeting, we found 33 genes related to SRP-dependent cotranslational protein targeting to membranes (7 in common, Figure 2E). As expected, those 33 genes are highly enriched in ribosome protein functions (29 genes associated, Figure 2F). In addition, 31 out from the 33 genes are related to protein translation (corrected *p*-value = 10 × 10^−56.43^) and seven genes are related to the VEGFA-VEGFR2 (vascular endothelial growth factor A–vascular endothelial growth factor receptor 2) signaling pathway (corrected *p*-value = 10 × 10^−3.76^). More importantly, these 33 genes are connected to each other and might form a strongly connected complex. In summary, independent technology and TNBC cohort-based cross validation helped us identify the important concordant copy number gain and upregulation events at the single-cell level. These consistent results from thousands of single cells also make the top ranked ribosome protein modules reliable.

### 2.3. The Mutational and Survival Analysis on 6688 Breast Cancer Samples in 15 Studies

**Figure 3:**
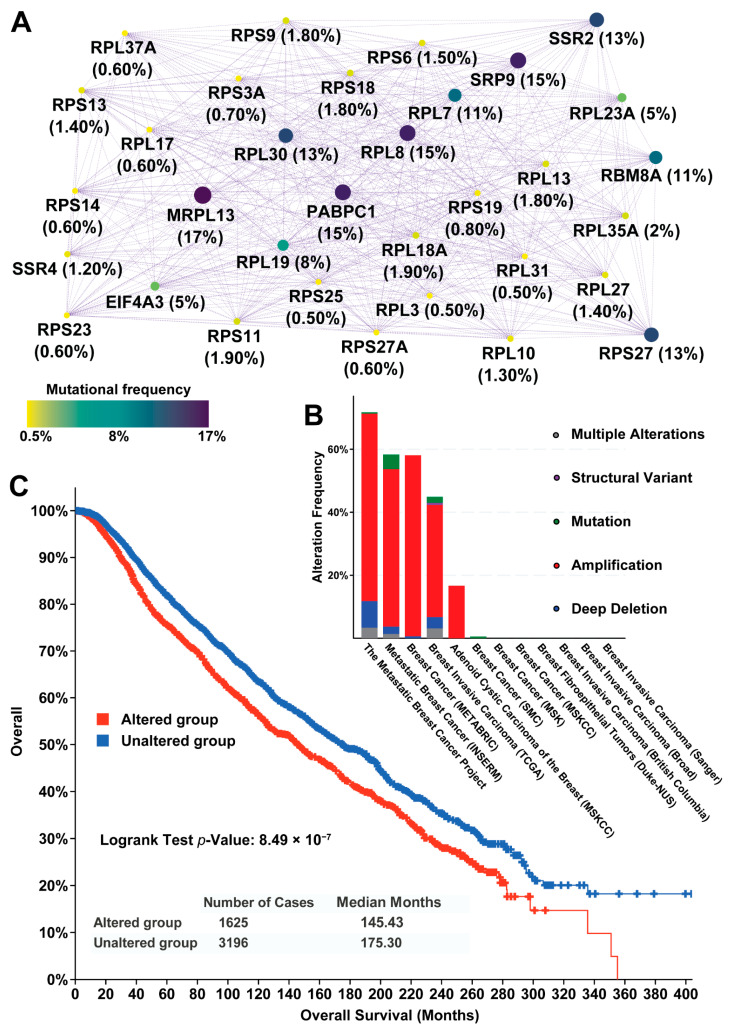
The overall mutational and prognostic features for 33 genes with increased gene expression induced by CNGs. (**A**) The gene–gene interaction network showing the mutational frequency in 6688 breast cancer samples combined from 12 studies. The size and color of each node is correlated with the number of connections. (**B**) The mutational summary for twelve breast cancer datasets; (**C**) The overall survival plot shows the median survival months for patients with or without mutations on the 33 genes.

By applying our computational framework, we identified several important somatic CNV features at the single-cell level, particularly with respect to the ribosome proteins and their effects on mRNA translation and sorting. We hypothesized that amplification-induced high expression activity of ribosome proteins may select for breast cancers that render them invasive and aggressive for metastasis. We therefore asked whether those 33 ribosome complex genes are frequently mutated in breast cancer and metastatic TNBC. As shown in Figure S1, we utilized a public cancer genomic resource and combined 6688 breast cancer samples with genetic mutation data from 12 independent studies. Since these 33 genes formed a strongly connected module, we organized the mutational frequency by interaction map (Figure 3A). Generally, the ribosome proteins with higher mutational frequency tended to have more connections in the network. Of the top ten mutated genes, nine were also connected with more genes, including MPRL13 (mitochondrial ribosomal protein L13, 17%), SRP9 (signal recognition particle 9, 15%), PABPC1 (poly(A) binding protein cytoplasmic 1, 15%), RPL8 (ribosomal protein L8, 15%), RPL30 (ribosomal protein L30, 13%), SSR2 (signal sequence receptor subunit 2, 13%), RPS27 (ribosomal protein S27, 13%), RBM8A (RNA-binding protein 8A, 11%), and RPL7 (ribosomal protein L7, 11%). In fact, the topological features, such as number of connections, were found to be associated with the mutational rates of the cancer driver genes [8].

Of the individual datasets and breast cancer subtypes (Figure 3B), three metastatic breast cancer cohorts were highly mutated (over 50% patients in the corresponding cohort). In contrast, another seven cohorts, which showed markedly lower mutational frequency, were not metastatic. In between, there were two datasets. One was TCGA invasive carcinoma and the other was adenoid cystic carcinoma of breast. These results may imply that the 33 genes related to contranslational proteins targeting to membranes are highly mutated in metastatic breast cancers but not in non-metastatic cancers.

More importantly, survival curves based on those 33 CNG-UP genes showed the significant difference among those combined 4821 breast cancer patients. In the overall survival analysis, patients were segregated into “altered group” (red line) and “unaltered group” (blue line). The 1625 patients with certain genetic mutations on these 33 genes had a median 145.43 survival months while those patients without any mutations lived 175.30 months as a median. The logrank test statistical *p*-value was 8.94 × 10^−7^, which was corrected to a Q-value = 4.24 × 10^−6^. Besides the overall survival result, another four survival analyses further confirmed the statistical difference between the two groups, including relapse-free survival (Q-value = 5.26 × 10^−4^), disease-specific survival (Q-value = 0.0416), disease-free survival (Q-value = 0.0488), and progression-free survival (Q-value = 0.0488). In summary, these results confirmed the 33 genes are important for cancer metastasis and patient prognosis.

**Figure 4:**
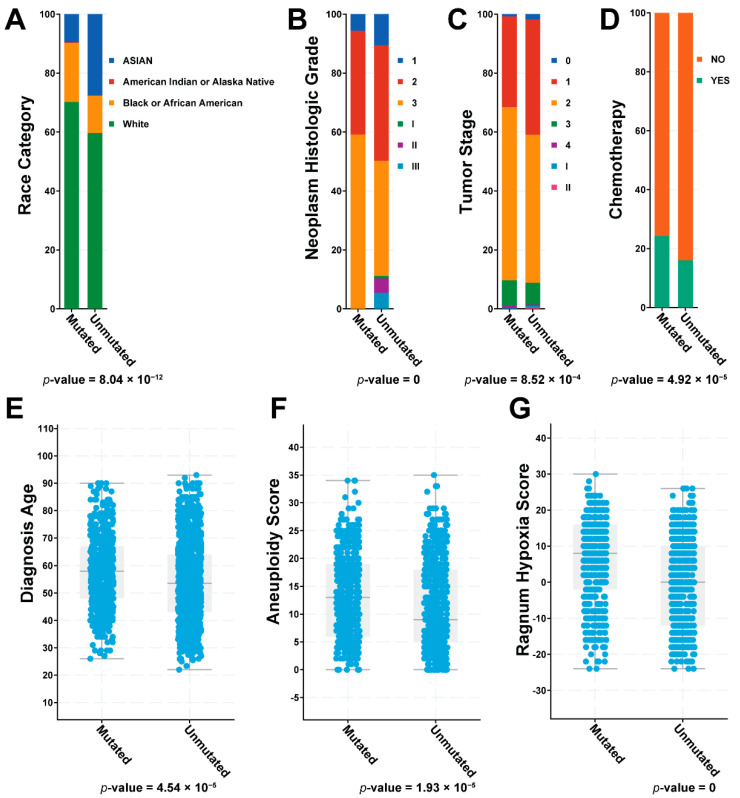
Clinical features summary for the 33 ribosome genes on (**A**) race category; (**B**) neoplasm histological grade; (**C**) tumor stage; (**D**) chemotherapy treatment; (**E**) diagnosis age; (**F**) aneuploidy score; and (**G**) ragnum hypoxia score. The *p*-values are the statistical tests between patients with or without any genetic mutations on the 33 genes.

Strikingly, these mutations were also positively associated with other important clinical features (Figure 4), including the race category, diagnosis age, histological grade, tumor stage, aneuploidy score, hypoxia score, and chemotherapy treatment. For example, there were relatively fewer Asian patients than White patients in the group with mutations (Figure 4A). For histological grade and tumor stage, those patients with mutations tended to be in the higher grades (Figure 4B) or late stages (Figure 4C). Chemotherapy was applied more often to patients without any mutations for these 33 genes (Figure 4D). In terms of diagnosis age, patients with mutations on these 33 genes tended to be diagnosed later (Figure 4E), which might explain their higher grades and later stages. The presence of mutations was also indicative of higher aggression in factors such as aneuploidy (Figure 4F) and hypoxia (Figure 4G). The further mutational analysis expanded our understanding of the clinical features on those 33 ribosome proteins identified by our computational framework.

### 2.4. Intratumor Heterogeneity and Its Relationship with Key Cell States at the Single-Cell Level

According to the gene dosage hypothesis there is a positive correlation between gene copy number and mRNA expression where protein abundance between different cells will lead to a higher level of heterogeneity. Generally, scRNASeq is used to characterize the intratumor heterogeneity, which means identifying the subpopulations of cells superficially similar to other homogenous cells. In fact, measures of intratumor heterogeneity could also be used to detect changes in cell states and subsequent impacts on intratumor heterogeneity. For example, the cell stemness state is one of the key attributes comprising self-renewal, cell differentiation, and resistance to chemotherapy treatment.

**Figure 5:**
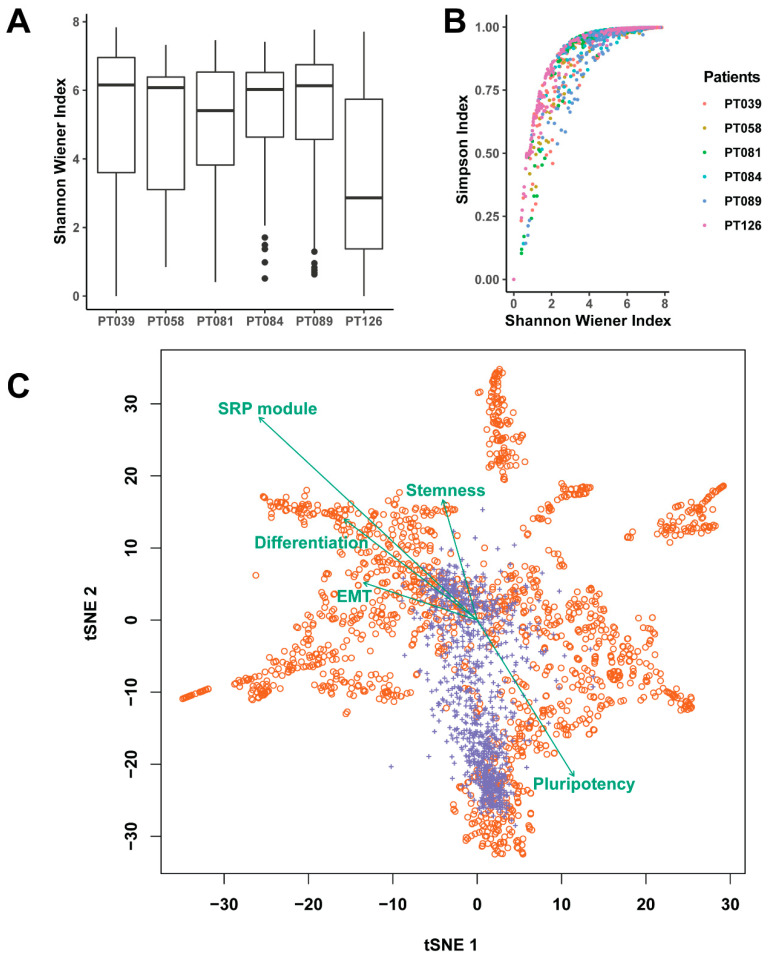
Intratumor heterogeneity and its relationship with five key cellular states. (**A**) The diversity based on the Shannon–Weiner index for six patients with hundreds of cells. (**B**) The correlation between Simpson index and Shannon–Weiner index in six patients; (**C**) The t-SNE biplots of cells (red) and genes (purple). The teal lines and labels correspond to the cell states’ vector. The orange circles represent cells and purple “+” are the genes ranked with the top 10% of variations.

As shown in Figure 5A, we only focused on the six patients from the primary dataset GSE118389. For each patient, we calculated two heterogeneous indices: Shannon–Wiener index (Figure 5A) and Simpson index (Figure S2). These two indices are the classic diversity indices in ecology that depict alpha diversity, which represents the species richness in a plot. In detail, the Shannon–Wiener index is a measure of diversity that combines a species’ richness and relative abundance in a plot, while the Simpson index is more about the dominance of the species as it accounts for the proportion of a species in a community. Here, both indices were used to characterize how the number of expressed genes (analogue to species) were expressed in a cell (analogue to ecological plot). In this way, each cell had an expression richness index-to-gene expression variation. By mixing all the cells from a patient, we had an overall heterogeneous feature of gene expression. As shown in Figure 5A, patient PT126 had the lowest Shannon–Wiener index, which means fewer genes were expressed, and those genes had lower expression levels in those cells in PT126. In Figure 5B, we describe the general trend between the two different indices. Both indices highly correlated to each other, which confirmed the expression variations in patients.

In addition to the application of two classic diversity indices in our dataset, we also used the t-distributed stochastic neighbor embedding (tSNE) method to visualize the variations in all the cells. Based on those well-studied marker genes, we further defined five cell states that could be used to describe the cellular microenvironment and explain the intratumor variation. Specifically, five cell states were defined as breast cancer stemness, pluripotency, differentiation, proliferation, and epithelial–mesenchymal transition (EMT)/metastasis. For example, we combined the expression of CD44 (cluster of differentiation gene 44), ITGA6 (integrin subunit alpha 6), DNER (delta/notch like EGF repeat containing), ALDH1A3 (aldehyde dehydrogenase 1 family member A3), and ABCG2 (ATP binding cassette subfamily G member 2) to characterize breast cancer stemness. Similarly, we also combined all of the 33 genes to describe the overall SRP-dependent cotranslational proteins targeting functions in all the cells (SRP module). By checking the relationship between our ribosome proteins and the five cell states (Figure 5C), we found those ribosome proteins presented positive associations with cell differentiation, stemness, and EMT/metastasis. However, we also observed huge differences at the patient level (Figure S3). For example, the SRP modules are not well correlated with other cell states in PT126 due to a lack of sufficient expression data. Together, these findings may imply the ribosome protein-based signatures can be useful to predict the cell stemness and differentiation states, which is important for cancer metastasis or EMT.

## 3. Discussion

Our objective in this study was to determine the gene dosage effect of low and high copy number in tumor cell communities. According to the dosage effect, we expected that copy number variation would affect both mRNA and protein abundance. Though our previous pan-cancer-based analyses on both tumor suppressors and oncogenes identified hundreds of critical gene expression changes consistent with dosage effect [4,5], the concordant CNV and the gene expression dosage were largely unknown at the single-cell level.

In this study, we presented the first systematic examination of the expression of quantitative traits that provides more depth in the knowledge about the relationship between CNV and gene expression at the single-cell level. By focusing on a metastatic TNBC cohort with matched CNV and scRNAseq profiling on 1534 single cells, we identified 47,198 consistent copy number gains (CNGs) and gene upregulation from 1145 single cells. By filtering out those concordant events detected in less than 100 distinct cells, we further scrutinized 94 genes with active amplification. Interestingly, the ribosome protein’s function for cotranslational protein sorting is enriched in these 94 genes.

Cross-validation between two datasets is highly recommended when running through our computational framework, although the numbers of sequenced cells may have substantial differences. When a validating dataset is not available, we recommend using the experimental approach to validate the potential gene dosage effect at the single-cell level. In our study, the independent dataset also confirmed the abundant amplifications on the ribosomal gene for cotranslational protein targeting. Strikingly, these genes were significantly associated with patient survival time based on thousands of breast cancer patients from bulk sequencing. Since ribosome proteins are the critical molecular machines for cell growth, driving cancer initiation and metastasis, the large-scale change of multiple ribosome proteins may promote protein synthesis. Therefore, ribosome protein could be used as the potential drug to suppress the metastasis of TNBC [9].

The main conclusion from this study was mainly based on the two independent TNBC datasets with matched WES-based CNV and scRNAseq-based transcriptome data. A potential limitation of this study is that we only focused on protein-coding genes based on the huge number of consistent CNG-UP events. More comprehensive genomics profiling for microRNAs and long non-coding RNAs at the single-cell level may provide new insights into potential regulatory roles associated with CNVs. Based on consistent results from hundreds of cells, we filtered out many low-frequency CNG-UP events, which might be noise during cell differentiation. Due to the droplet problem in single-cell sequencing technology [10], another limitation may be the loss of some signals for both CNV and transcripts. These undetected CNVs and their expression may also contribute to TNBC progression and metastasis. Additionally, we intend to explore the knowledge gap of ribosome protein amplification at the single-cell level and therefore no attempt was made to explore the gene effect on subpopulation colonization. Lastly, the dynamic changes of gene expression can differ from one patient to another. That leads to an important interaction between changes in gene expression observed in one patient across different time points and differences between patients. There is a need to develop models and theories based on this variability to improve tumor subpopulation management based on various clinical features.

To expand our understanding about the clinical features on the ribosome proteins, we defined the ribosome cell state based on the 33 genes. By correlating the ribosome state and other cell states such as stemness, we explored the potential application of making ribosome proteins critical targets in cancer therapy [11]. While ribosome protein function is associated with key cell states such as stemness, the ribosome protein-based state may indirectly correlate with stemness in some individuals due to a lack of expression data. As the controller of protein synthesis and sorting in cells, ribosome proteins are important drivers of most cellular events. Therefore, the dosage effect of ribosome proteins may disrupt the whole cellular molecular ecosystem or population structure and change metabolite and energy availability. The amplification of ribosome proteins could shift protein assembly dynamics from deterministic to stochastic. Those cellular stochastic processes, both at the mRNA and protein levels, influence the function and progression of cancer. As disturbances of ribosome proteins increase in frequency and intensity, the tumor cells may become more invasive, and metastasis will be favored. Therefore, therapeutically targeting the ribosome machine may restore the cell’s function.

## 4. Materials and Methods

To explore the gene dosage effect at the single-cell level, we proposed a computational framework to determine the concordant expression change and copy number variations.

### 4.1. The Two TNBC Datasets with Independent CNV and scRNAseq Data

To conduct a systematic CNV survey in metastatic TNBC, we searched the gene expression omnibus (GEO) and PubMed databases [12]. After screening the sample and data information manually, we found two datasets that satisfied the criteria with both CNV and expression profiles at the single-cell level. For the first primary dataset GSE118389, there were 1530 cells from six patients, which was ideal for our designed computational framework. The other study GSE75688 was conducted in 2017 and the number of cells was comparatively smaller; we used this as the validating dataset.

To map those CNVs to human genes, we downloaded genomic locations of all the human genes from the NCBI RefSeq database [12]. By applying Bedtools [13], all the genomic locations were mapped to the characterized CNV genomic coordinates. In the primary dataset, 20,190 genes were annotated with accurate CNVs based on their overlapped genomic locations. For the validating dataset, 43,739 CNVs were detected and were associated with 21,686 genes.

### 4.2. The Gene Expression Change for the Matched Tumor Samples with CNV

To explore the gene dosage effect, expression profiles across all the cells were used to compute up/downregulation. In practice, two expression profiles were downloaded from the corresponding GEO pages. To quantify the expression, we adopted TPM instead of FPKM, which can calculate a different sum of the normalized reads in each cell. In contrast, the sum of all TPMs in each cell are the same, which makes it unbiased for comparing expressions in different cells [14]. Based on our framework, we only focused on the direction of gene expression changes in a specific cancer cell. Therefore, we did not run the differential expression analyses across different cell types. Rather, we computed the average and the standard deviation of expression for a gene across all the cells. The Z-score was utilized to examine whether a gene was over or under-expressed in a specific cell as described in our previous publication [4]. The equation for Z-score is as follows:

Z = (x − µ)/σ,(1)

The x is the expression value in a specific cell; µ is the mean expression value of the gene across all cells; and σ is the standard deviation of the gene expression across all cells.

Theoretically, each expression score should be transformed to a Z-score. Due to the droplet issue in single-cell RNA sequencing, many expression scores were recorded as zero, which does not always imply the real expression status, but rather a technical barrier. Therefore, we did not use the Z-scores if their original expression values were zero. Then, we set a threshold of Z-scores with absolute values over 1.96 to scrutinize those cells whose genes’ Z-scores were satisfied with the 1.96 cut-off. Positive Z-scores higher than 1.96 were defined as upregulation, while the Z-scores under −1.96 were grouped as downregulation. By matching the Z-score for each gene, we mapped the gene up/downregulation to the CNV data in the same patients.

### 4.3. Functional Module Identification and Mutational Features for the Genes in the Top-Ranked Module

The MCODE module was identified based on the online gene–gene interaction platform PINA 3.0 [15]. The inputs were those genes with concordant CNV and expression changes in multiple cells. The function for those top-ranked modules was further explored by R package gprofiler2 [16]. For those 33 SRP-related genes belonging to the same functional module, we further inspected their somatic mutational profile in 12 publicly available breast cancer datasets using the cBio portal [17]. In the cBio portal, we explored the sample-based oncoprint and also made a summary based on the dataset. In addition, we combined all the samples and explored the patients’ clinical features and survival curves.

### 4.4. Cell States, Diversity Indices, and Dimensional Reduction Analysis

To characterize cell states for each cell, the R package GSVA [18] was used. The five cell states were defined based on the previously published research about the identification of distinct breast cancer stem cell populations [19]. In practice, the genes (Figure 1E from [19]) were used as the signatures to characterize EMT, stemness, pluripotency, differentiation, and proliferation. Those key markers are specific for EMT/metastasis (*SNAI1*, *SNAI2*, *FOSL1*, *VIM*, *CDH2*, *ID1*), breast cancer stemness (*CD44*, *ITGA6*, *DNER*, *ALDH1A3*, *ABCG2*), pluripotency (*POU5F1*, *NANOG*, *SOX2*), differentiation (*CDH1*, *CD24*, *EPCAM*, *ESR1*, *PGR*) and proliferation (*CCNA2*, *MKI67*, *ERBB2*). In order to explore the cell state about SRP-dependent protein synthesis and sorting, we used the 33 genes as signatures to quantitate the cell state related to the SRP module. To explore if those states correlated with the heterogeneity in the dataset, we used t-SNE biplots for the GSE118389 dataset and six patients individually. By adding the vector with arrows in the t-SNE chart, we displayed those cell states with direction and quantity. The directions of the vectors were towards where the cell state vectors changed most rapidly. The lengths of those vectors were automatically adjusted for combination of all signatures.

To further explore the tumor heterogeneity, we adopted the equations for the Shannon–Wiener index [20] for a cell:

(2) $$H\;=\;-\sum_{i=1}^{S}p_{i}\left(ln\;p_{i}\right)$$

Another tumor heterogeneity value at the single-cell level is based on the Simpson index [20] for a cell:

(3) $$D\;=\;1\;-\;\sum_{i=1}^{S}p_{i}$$

In both equations, the $p_{i}$ is the proportion of the expressed gene *i* in a specific cell. Therefore, *S* is the number of expressed genes and $\sum_{i=1}^{S}p_{i}$ = 1.

R package Seurat [21] was used to calculate the dimensionality reduction of t-SNE. The correlation between the first two dimensions of t-SNE and cell state vectors and the heterogeneity indices were estimated using the R package Vegan (https://github.com/vegandevs/vegan (accessed on 6 June 2021)).

## 5. Conclusions

In conclusion, we examined the gene dosage hypothesis at the single-cell transcriptome level. The results highlighted concordant copy number gain and gene upregulation. The genes affected by the dosage effect were enriched in SRP-dependent cotranslational proteins targeting membranes in two independent TNBC datasets. In addition, we found these ribosome genes could form highly connected functional modules and were highly related to patients’ survival. We also revealed that the amplification of ribosome proteins is enriched in metastatic breast cancers. Taken together, these results provide a deep insight into mechanisms of functional consequences of oncogenic ribosome protein copy number gains in breast cancer development.

## Data Availability

The raw data used were from public Gene Expression Omnibus databases GEO118390 and GSE75688.

## Supplementary Materials

The following are available online at https://www.mdpi.com/article/10.3390/ph14090918/s1, Figure S1: The mutational profile for 94 genes in 6688 breast cancer samples, Figure S2: The Simpson index based on the single-cell gene expressions from six patients, Figure S3: Intratumor heterogeneity in six patients visualized by t-SNE biplots of cells (red) and genes (purple). The teal lines and labels correspond to the cell states’ vector. The orange circles represent cells and “+” are the genes ranked as the top 10% of variations, Table S1: The 89,524 consistent CNV-expression events with absolute expression Z-scores over 1.96, Table S2: The five distinct functional modules identified by molecular complex detection (MCODE).
