# Supplementary figures and images for: Gene Dosage Analysis on the Single-Cell Transcriptomes Linking Cotranslational Protein Targeting to Metastatic Triple-Negative Breast Cancer
Source: Pharmaceuticals (Basel). 2021 Sep 10;14(9):918. doi: 10.3390/ph14090918 (PMC8472593; doi:10.3390/ph14090918)

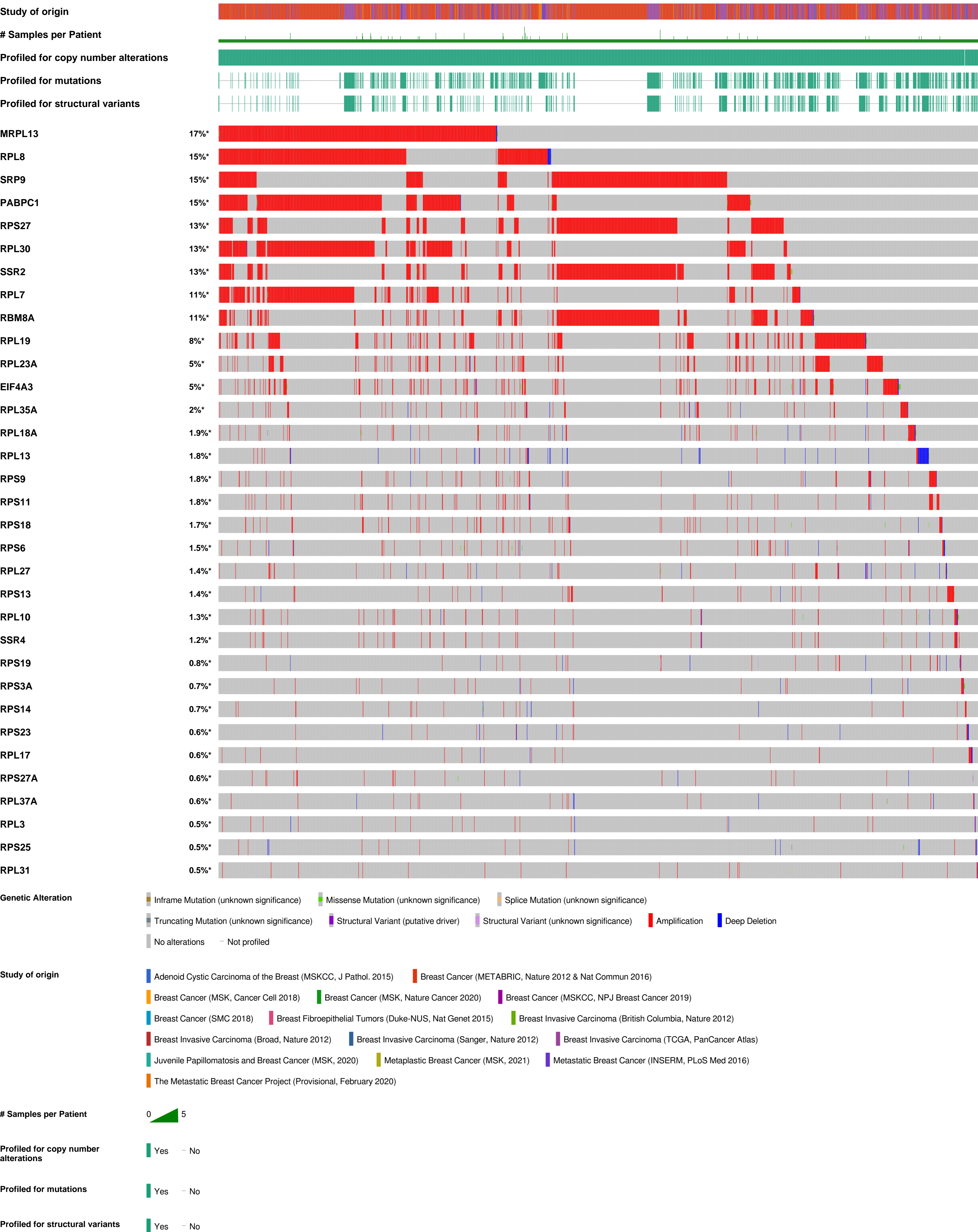

Supplement: Supplementary file 1 [file pharmaceuticals-14-00918-s001.zip › Supp_submit/FigureS1_oncoprint_33genes.pdf]

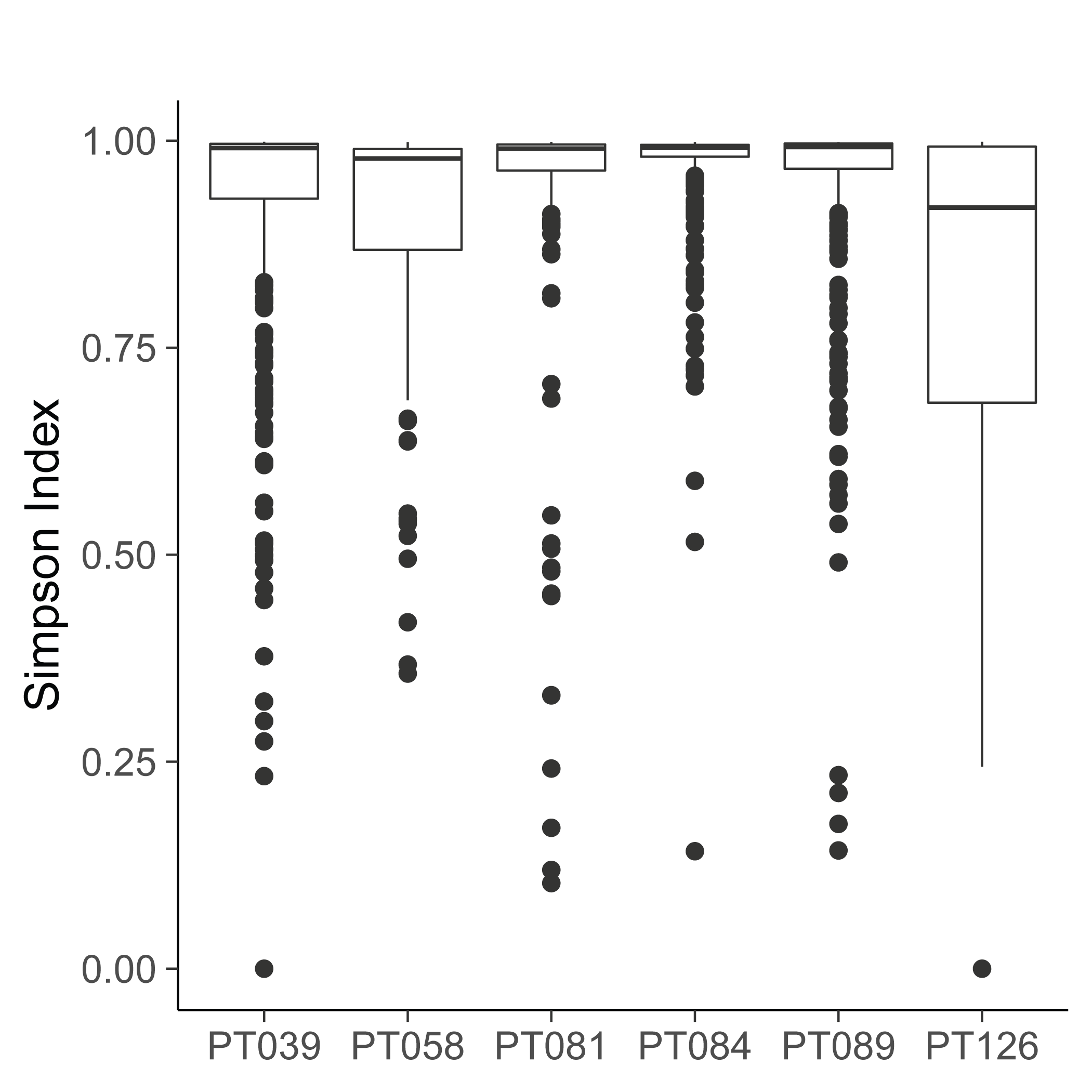

Supplement: Supplementary file 1 [file pharmaceuticals-14-00918-s001.zip › Supp_submit/FigureS2_simpson_index.tif]

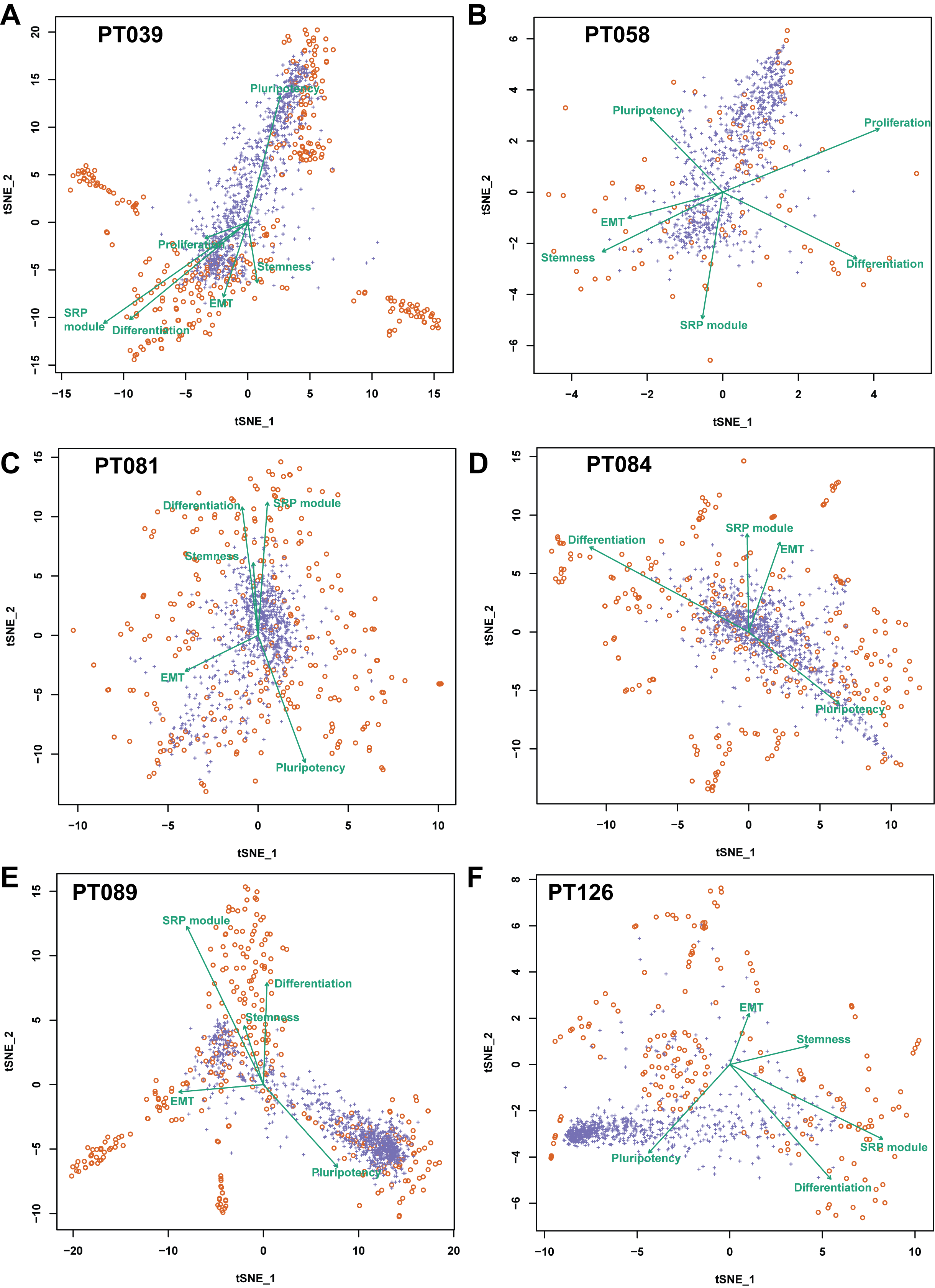

Supplement: Supplementary file 1 [file pharmaceuticals-14-00918-s001.zip › Supp_submit/FigureS3_tSNE.tif]
